# Morphophysiological and biochemical response of mungbean [Vigna radiata (L.) Wilczek] varieties at different developmental stages under drought stress

**DOI:** 10.3906/biy-1801-64

**Published:** 2019-02-07

**Authors:** Pooja BANGAR, Ashok CHAUDHURY, Bhavana TIWARI, Sanjay KUMAR, Ratna KUMARI, Kangila Venkataramana BHAT

**Affiliations:** 1 Genomic Resources Division, ICAR-National Bureau of Plant Genetic Resources, Pusa Campus , New Delhi , India; 2 Guru Jambheshwar University of Science and Technology , Hissar , India

**Keywords:** Drought stress, membrane stability index, mungbean, orphophysiological traits, proline content, relative water content

## Abstract

The present study was conducted to assess the morphophysiological and biochemical responses during different developmental stages in mungbean varieties subjected to drought stress, and to screen the varieties for drought tolerance. A field experiment was performed according to a completely randomized design on 25 mungbean varieties with 3 replicates per variety. Stress treatment was applied at 3 levels: control (no stress), vegetative stage (25 days after sowing), and reproductive stage (35 days after sowing). According to combined analysis of variance, there were significant effects from drought stress on relative water content (RWC), membrane stability index (MSI), protein and proline content of leaves, leaf area, plant height, and yield traits. MSI, RWC, protein content, leaf area, plant height, and yield traits were decreased during drought stress, while proline content was increased under drought stress conditions. The results showed that the vegetative stage was more sensitive to drought stress, which was further supported by correlation analysis. Taken together, Vigna sublobata, MCV-1, PLM-32, LGG-407, LGG-450, TM-96-2, and Sattya varieties were identified as drought tolerant as they maintained the higher values of RWC, MSI, protein, proline content, leaf area, plant height, and yield traits. These varieties could be used in breeding programs for better physiological drought tolerance traits.

## 1. Introduction

Among abiotic stresses, drought stress is undoubtedly one of the most devastating environmental stresses. Drought is a multidimensional complex stress, simultaneously disturbing the physiological, morphological, biochemical, and molecular states which control the growth and quality of the crop and ultimately crop productivity (Basu et al., 2016) . This situation has been aggravated worldwide as drought-stressed areas are expanding rapidly due to uneven rainfall, limited water sources, and other rapid and drastic changes in global environmental conditions (Fahad et al., 2017) .

Mungbean [Vigna radiata (L.) Wilczek], alternatively known as the moong bean, green gram, or mugda is the third most important pulse crop after chickpea and pigeon pea. It is a diploid (2n = 22), self-pollinating, and fast-growing (<60 days) grain legume belonging to the family Fabaceae. Being a short-duration legume, it is an ideal legume for catch cropping, intercropping, and relay cropping. Mungbean has the ability to fix nitrogen via symbiosis with nitrogen-fixing Rhizobium bacterium (Allito et al., 2015). It is an excellent and easily digestible source of protein for humans where people are vegetarian (such as in India) or where meat is lacking, ultimately supporting food security. The raw and mature seeds are rich in nutrients including carbohydrates, protein, fibers, minerals, antioxidants like flavonoids (Quercetin-3-Oglucoside), and phenolics (Guo et al., 2012) . Despite being an economically important crop, overall production of mungbean is low due to abiotic and biotic stresses (Bangar et al., 2018) .

Mungbean grows mainly in rain-fed conditions at high temperatures (27–30 °C), with low humidity and moderate rainfall ranging from 60 to 80 cm. Due to this, it faces drought at different developmental stages. It is believed that mungbean thrives under drought conditions (Dutta and Bera, 2008; Ahmad et al., 2015) . However, mungbean varieties respond variably to drought stress depending on stress duration, growth stage, and variety of the crop. Various studies have shown variability in morphophysiological traits for drought tolerance among mungbean varieties during different developmental stages of growth (Naresh et al., 2013; Uddin et al., 2013) . Drought is the most important limiting factor for mungbean production. Due to the ongoing situation of water supplies for agriculture retreating, there is an urgent need to screen the drought tolerant varieties (Pandey and Sukla, 2015) . Several morphophysiological and biochemical parameters have been established for drought stress tolerance assessment in plants based on proline accumulation, high relative water content, leaf area index, yield components, antioxidant enzymatic activities, PEG mediation, etc. (Mafakheri et al., 2010; Almeselmani et al., 2011; Ranawake et al., 2012; Alderfasi et al., 2017; Swathi et al., 2017) . Therefore, to design an effective phenotypic screening strategy for crop improvement, a better understanding of the responses of mungbean varieties under different drought stress conditions is required (Abenavoli et al., 2016) . Further assessment of variable parameters and their correlation under drought conditions would be helpful in selecting diverse valuable varieties with defined growth strategies, which may be useful in breeding programs focused on drought tolerance (Sarkar et al., 2013; Abraha et al., 2015; Mishra and Panda, 2017; Tiwari et al., 2018) .

With this aim, this study was performed to understand the effects of drought stress on mungbean varieties at different developmental stages, i.e. vegetative and reproductive, on the basis of morphophysiological and biochemical traits. Correlation analysis was done among the morphophysiological attributes under drought stress conditions.

## 2. Materials and methods

The present study was conducted from April to June 2017 at the National Bureau of Plant Genetic Resources (NBPGR), Pusa Campus in New Delhi, India (28.080°N, 77.120°E) with temperatures ranging from ±30 to ±48 °C. The experiment was performed with 25 mungbean varieties with 3 replicates per variety according to the completely randomized design. Initially, the land was watered to near field capacity, and then plowing and harrowing was done thoroughly to a fine tilt before defining the plots. No chemical fertilizers or pesticides were used. Seeds were sown manually with plant to plant spacing of 10 cm and interrow spacing of 30 cm. The first hand-weeding was done 15 days after sowing and subsequent weeding was done regularly when needed. No machinery was used in this process. The experiment was done at 3 levels of treatment: no stress (control), stress at the vegetative stage (25 days after sowing) by removal of irrigation for 15 days, and stress at the reproductive stage (35 days after sowing) by removal of irrigation for 15 days. Drought stress was maintained by creating temporary rain sheds to avoid rainfall.

### 2.1. Physiological analysis

Relative water content (RWC) of the leaf was measured as described by Barrs and Weatherley (1962) for different treatments. Briefly, a 100-mg fully expanded leaf sample was taken and kept in distilled water for 4 h in a Petri plate at room temperature. The turgid weight of the samples was recorded. Samples were then oven-dried at 65 °C for 24 h and their dried weight was observed. RWC was calculated as follows: RWC (%) = [(FW – DW) / (TW – DW)] × 100; where FW = fresh weight, DW = dry weight, and TW = turgid weight.

Membrane stability index (MSI) for different treatments was calculated by recording electrical conductivities (Sairam, 1994) . The 100-mg leaf samples were put into 2 sets of test tubes containing 10 mL of distilled water. One set was heated at 40 °C for 30 min and the other set was heated at 100 °C for 10 min. Their electrical conductivities (C1 and C2) were recorded, respectively. MSI was calculated as follows: MSI (%) = 1 – (C1/C2) × 100; where C1 and C2 were electrical conductivity at 40 and 100 °C, respectively.

Leaf area was measured using a CI-203 laser area meter (CI-203, CID Inc., Camas, WA, USA) on the fully expanded leaves. Leaf chlorophyll content (SPAD index) was measured using a SPAD-502 chlorophyll meter (Minolta Corp., Ramsey, NJ, USA).

### 2.2. Biochemical analysis

Proline content was calculated by using the acid ninhydrin method (Bates et al., 1973) for both stage treatments. Proline was extracted from 100-mg fresh leaf samples in 2 mL of 3% aqueous sulphosalicylic acid, and absorbance was measured at 520 nm using toluene as the blank. Proline content was calculated using a standard curve and is expressed as µM/g.FW.

Protein content at both stages was estimated by Bradford’s method (1976 ); 100-mg leaf samples were homogenized in phosphate buffer and measured spectrophotometrically at 595 nm against a reagent blank.

### 2.3. Morphological analysis

All measurements were taken from 3 healthy randomly chosen plants for all treatments. Plant height was measured by a scale from the soil surface to the highest tip of the plant. The yield-defining parameters like number of pods per plant, number of pods per cluster, number of clusters per plant, number of seeds per pod, and 100 seed weight were measured and recorded after harvesting.

### 2.4. Statistical analysis

Morphophysiological data were subjected to two-way analysis of variance (ANOVA) using GenStat (8th edition) to determine the significance of the results among genotypes, different treatments, and the interaction effect between genotype and treatments. Mean values were calculated from 3 replicates using standard error of mean. A correlation analysis was performed to study the relationship between multiple traits. A dendrogram based on Manhattan distance was constructed according to the unweighted pair-group mean arithmetic method (UPGMA) using Numerical Taxonomy System software, version 2.1 (NTSYSpc, Exeter Software, Setauket, NY, USA) with V. sublobata as an outgroup for rooting the tree.

## 3. Results and discussion

Among the 25 mungbean varieties, all of the traits exhibited significant variation (P < 0.001 and P < 0.05) (Table 1). The mean results showed a significant decrease in physiological traits such as RWC and MSI in all varieties when exposed to drought conditions at both the vegetative (Table 2) and the reproductive (Table 3) stages when compared to control conditions. The decrease in MSI is probably due to the fact that under drought conditions the overproduction of reactive oxygen species occurs, which disrupts the cell membrane by altering its phospholipid and fatty acid compositions (Sibel and Birol, 2007; Ratnasekera and Subhashi, 2015) . Under drought conditions, MSI ranged from 74.23% to 91.23% with a mean of 86.54% during the vegetative stage, and ranged from 47.85% to 85.10% with a mean of 67.74% during the reproductive stage.

**Table 1:** Mean squares of combined analysis of variance from morphophysiological and biochemical traits during both stages under drought stress.

Stage	Veg	Rep	Veg	Rep	Veg	Rep	Veg	Rep
Source of variation (D.F)	Genotype (24)		Treatment (1)		Genotype × Treatment (24)		Residuals (98)	
RWC	161.22**	47.71**	2215.05**	1054.22**	29.91**	19.29**	0.58	0.32
MSI	41.90**	648.54**	1033.38**	5529.50**	28.70**	66.18**	0.14	0.49
Protein	4.05**	20.06**	64.62**	195.52**	1.35**	5.31**	0.003	0.05
Proline	0.45**	0.86**	18.07**	23.62**	0.20**	0.46**	0.003	0.002
CC	45.86**	54.08**	285.94*	58.66*	23.55**	73.09**	11.02	7.64
Plant height	553.73**	497.86**	846.33**	245.76**	4.83**	2.78**	0.40	1.12
Leaf area	1568.53**	2041**	38,757.69**	16,730**	495.53**	296.4**	0.10	0.05
100 seed weight	0.38**	0.30**	3.09**	0.48**	0.03**	0.007**	0.001	0.001
No. of seeds/pod	5.31**	5.39**	204.17**	83.63**	1.72*	0.97ns	0.78	0.81
No. of pods/cluster	0.73**	0.67**	76.33**	36.51**	0.24ns	0.30ns	0.26	0.36
No. of pods/plant	2.92**	3.05**	213.61**	152.007**	0.82*	0.74ns	0.49	0.53
No. of clusters/plant	3.11**	3.14**	179.31**	142.11**	0.56ns	0.78*	0.39	0.41

* Significant at 5%; ** significant at 1%.

Veg = Vegetative, Rep = Reproductive, D.F = Degree of freedom, No. = Number, RWC = Relative water content, MSI = Membrane stability index, CC = chlorophyll content.

**Table 2:** Mean values for RWC, MSI, protein, proline, and chlorophyll content during vegetative stage under drought stress.

Varieties	RWC			MSI			Protein			Proline			CC	
	Varieties	Con.	Str.	% dec	Con.	Str.	% dec	Con.	Str.	% dec	Con.	Str.	% inc	Con.	Str.
V. sublobata	76.09 ± 0.37	71.30 ± 0.39	6.3	94.61 ± 0.37	90.37 ± 0.39	4.5	3.33 ± 0.01	2.37 ± 0.06	29	2.17 ± 0.02	2.87 ± 0.06	31.9	42.63 ± 1.53	48.10 ± 2.70
Asha	83.27 ± 0.47	75.95 ± 0.36	8.8	87.70 ± 0.47	85.55 ± 0.36	2.5	3.91 ± 0.01	2.85 ± 0.13	27.1	2.15 ± 0.01	2.88 ± 0.02	33.8	41.67 ± 1.48	40.93 ± 1.22
DFS-8	77.96 ± 0.39	76.44 ± 0.45	2.0	92.48 ± 0.39	91.23 ± 0.45	1.4	5.10 ± 0.01	3.62 ± 0.01	29.1	1.81 ± 0.01	2.28 ± 0.03	26.2	38.83 ± 1.24	41.17 ± 1.41
Kopergaon	75.48 ± 0.36	69.63 ± 0.91	7.8	89.65 ± 0.36	88.85 ± 0.91	0.9	4.08 ± 0.03	2.38 ± 0.03	41.8	1.82 ± 0.01	2.15 ± 0.01	18.5	32.27 ± 1.27	42.90 ± 1.34
TARM-1	76.90 ± 0.45	61.89 ± 0.56	19.5	92.46 ± 0.45	89.46 ± 0.56	3.2	4.14 ± 0.03	1.59 ± 0.02	61.8	2.09 ± 0.03	2.24 ± 0.01	7.5	41.37 ± 1.42	43.53 ± 1.20
K-851	76.09 ± 0.38	73.16 ± 0.51	3.9	94.04 ± 0.38	89.55 ± 0.51	4.8	3.62 ± 0.02	3.55 ± 0.01	2.1	2.15 ± 0.07	2.29 ± 0.01	6.5	42.07 ± 0.76	42.40 ± 0.67
LGG-407	85.71 ± 0.33	71.35 ± 0.49	16.8	93.47 ± 0.33	87.89 ± 0.49	6.0	2.92 ± 0.02	2.90 ± 0.03	0.73	1.71 ± 0.01	2.11 ± 0.03	23.7	41.80 ± 2.19	40.73 ± 2.94
MGG-351	83.83 ± 0.36	80.76 ± 0.37	3.7	91.47 ± 0.36	86.18 ± 0.37	5.8	3.46 ± 0.03	1.73 ± 0.01	50.2	1.86 ± 0.03	2.51 ± 0.01	34.9	46.77 ± 1.99	42.03 ± 1.77
MCV-1	86.01 ± 0.33	84.44 ± 0.34	1.8	91.88 ± 0.33	90.61 ± 0.34	1.4	4.23 ± 0.03	1.84 ± 0.01	56.5	2.73 ± 0.03	3.41 ± 0.01	25.0	44 ± 2.09	41.37 ± 0.44
PAU-911	94.93 ± 0.48	84.89 ± 0.39	10.6	90.58 ± 0.48	89.91 ± 0.39	0.7	4.72 ± 0.03	2.85 ± 0.03	39.6	1.69 ± 0.01	2.35 ± 0.03	38.8	45.93 ± 0.90	44.20 ± 2.64
PDM-11	78.28 ± 0.08	74.91 ± 0.42	4.3	92.81 ± 0.08	84.84 ± 0.42	8.6	5.35 ± 0.02	2.42 ± 0.01	54.8	1.53 ± 0.02	2.40 ± 0.01	56.8	37.53 ± 1.59	47.10 ± 3.18
Phule-m-2	83.66 ± 0.61	79.86 ± 0.23	4.5	90.25 ± 0.61	74.23 ± 0.23	17.7	2.85 ± 0.03	1.38 ± 0.01	51.7	1.96 ± 0.02	3.25 ± 0.03	65.4	35.77 ± 1.87	46.43 ± 2.51
Samrat	85.20 ± 0.30	69.96 ± 0.12	17.9	93.52 ± 0.30	90.36 ± 0.12	3.4	3.31 ± 0.01	1.73 ± 0.01	47.9	1.73 ± 0.02	2.89 ± 0.03	67.0	43.47 ± 0.28	48.17 ± 2.06
Sattya	86.87 ± 0.23	75.03 ± 0.12	13.6	92.54 ± 0.23	87.25 ± 0.12	5.7	3.80 ± 0.01	3.54 ± 0.03	6.85	1.92 ± 0.02	2.40 ± 0.01	24.9	42.30 ± 1.56	47.47 ± 1.92
TM-96-2	82.54 ± 0.36	74.34 ± 0.51	9.9	93.44 ± 0.36	89.17 ± 0.51	4.6	2.11 ± 0.03	1.84 ± 0.01	12.9	1.72 ± 0.04	3.09 ± 0.04	80.2	44.20 ± 1.01	46.43 ± 1.79
WGG-37	74.88 ± 0.35	68.88 ± 0.91	8.0	89.09 ± 0.35	87.32 ± 0.91	1.98	1.68 ± 0.01	1.46 ± 0.02	13	1.94 ± 0.03	3.11 ± 0.01	60.0	47.57 ± 1.76	50.40 ± 4.11
LGG-460	83.59 ± 0.34	70.29 ± 0.37	15.9	91.30 ± 0.34	87.19 ± 0.37	4.5	2.73 ± 0.02	1.68 ± 0.01	38.3	1.81 ± 0.01	2.35 ± 0.03	29.6	40.20 ± 2.16	41.47 ± 1.95
PDM-54	73.87 ± 0.21	63.97 ± 0.47	13.4	94.58 ± 0.21	80.43 ± 0.47	14.9	2.81 ± 0.01	1.85 ± 0.01	34	2.14 ± 0.05	3.13 ± 0.05	46.3	44.50 ± 1.90	46.13 ± 0.68
SML-32	84.13 ± 0.55	78.19 ± 0.41	7.0	91.56 ± 0.55	84.11 ± 0.41	8.1	1.85 ± 0.03	1.63 ± 0.01	12.3	2.11 ± 0.04	2.31 ± 0.05	9.8	40.70 ± 1.99	39.93 ± 2.27
IC-10492	74.86 ± 0.58	67.16 ± 0.26	10.3	88.68 ± 0.58	83.69 ± 0.26	5.6	4.10 ± 0.05	2.69 ± 0.01	34.5	1.67 ± 0.03	2.73 ± 0.01	63.6	40.53 ± 2.58	45.57 ± 0.55
PLM-32	92.06 ± 0.61	78.19 ± 0.51	15.1	88.64 ± 0.61	88.36 ± 0.51	0.3	4.55 ± 0.03	4.04 ± 0.03	11.3	1.71 ± 0.01	2.66 ± 0.03	56.0	42.20 ± 2.35	42.83 ± 2.37
IPM-02-03	81.34 ± 0.34	73.09 ± 0.82	10.1	92.83 ± 0.34	89.12 ± 0.82	4.0	4.08 ± 0.06	3.20 ± 0.01	21.6	1.88 ± 0.03	2.61 ± 0.01	38.7	37.23 ± 2.41	43.40 ± 1.97
LGG-450	73.33 ± 0.08	71.31 ± 0.37	2.8	92.78 ± 0.08	78.67 ± 0.37	15.2	5.93 ± 0.06	2.25 ± 0.01	62	2.42 ± 0.01	2.67 ± 0.03	10.6	41.13 ± 3.62	39.10 ± 1.22
PM-5	86.79 ± 0.06	75.34 ± 0.48	13.2	94.36 ± 0.06	90.47 ± 0.48	4.1	2.36 ± 0.02	1.68 ± 0.01	28.6	2.17 ± 0.08	3.17 ± 0.04	46.0	48.83 ± 3.0	43.20 ± 0.70
PDM-139	80.09 ± 0.29	75.26 ± 0.69	6.0	89.97 ± 0.29	78.68 ± 0.69	12.5	3.45 ± 0.01	1.64 ± 0.01	52.5	2.10 ± 0.01	2.48 ± 0.01	17.8	43.17 ± 1.39	41.70 ± 0.53
Generalmean	81.50 ± 0.36	73.82 ± 0.46	9.3	91.79 ± 0.16	86.54 ± 0.19	5.7	3.62 ± 0.02	2.35 ± 0.02	32.8	1.96 ± 0.03	2.65 ± 0.02	36.8	41.87 ± 1.77	43.87 ± 1.77
Minimum	73.33	61.89	1.8	87.70	74.23	0.3	1.68	1.38	0.73	1.53	2.11	6.5	32.27	39.10
Maximum	94.93	84.89	19.5	94.61	91.23	17.7	5.93	4.04	62.0	2.73	3.41	80.2	48.83	50.40
Std. dev	0.62	0.79		0.28	0.33		0.04	0.03		0.04	0.04		3.07	3.06

Con. = Control, Str. = Stress, inc = Increase, dec = Decrease, RWC = Relative water content, MSI = Membrane stability index, and CC = chlorophyll content.

**Table 3:** Mean genotype values for RWC, MSI, protein, proline, and chlorophyll content during the reproductive stage under drought stress.

Varieties	RWC			MSI			Protein			Proline			CC	
	Varieties	Con.	Str.	% dec	Con.	Str.	% dec	Con.	Str.	% dec	Con.	Str.	% inc	Con.	Str.
V. sublobata	78.82 ± 0.37	77.66 ± 0.23	1.5	85.46 ± 0.18	83.19 ± 0.15	2.7	7.73 ± 0.02	6.03 ± 0.06	21.9	2.27 ± 0.01	2.82 ± 0.05	23.9	41.73 ± 0.88	49.77 ± 0.78
Asha	80.51 ± 0.17	78.31 ± 0.46	2.7	90.23 ± 0.57	78.15 ± 0.41	13.4	6.53 ± 0.01	4.57 ± 0.24	30.0	2.48 ± 0.01	2.92 ± 0.01	17.9	42.70 ± 1.42	43.00 ± 1.76
DFS-8	76.36 ± 0.18	72.87 ± 0.25	4.6	76.01 ± 0.20	61.07 ± 0.57	19.7	6.52 ± 0.15	4.72 ± 0.04	27.6	2.33 ± 0.01	2.56 ± 0.01	9.9	41.77 ± 2.77	48.40 ± 0.98
Kopergaon	76.92 ± 0.54	70.94 ± 0.35	7.8	89.28 ± 0.36	72.30 ± 0.13	19.0	10.41 ± 0.19	6.50 ± 0.08	37.6	1.64 ± 0.02	2.07 ± 0.03	26.5	36.90 ± 1.97	43.07 ± 1.85
TARM-1	80.21 ± 0.04	74.79 ± 0.26	6.8	78.22 ± 0.46	53.87 ± 0.57	31.1	10.75 ± 0.35	5.72 ± 0.20	46.8	2.12 ± 0.01	2.33 ± 0.02	9.6	41.93 ± 1.79	42.73 ± 1.15
K-851	77.88 ± 0.21	69.96 ± 0.37	10.2	88.65 ± 0.26	85.10 ± 0.46	4.0	6.18 ± 0.05	4.67 ± 0.06	24.4	2.23 ± 0.04	2.46 ± 0.01	10.1	46.13 ± 5.41	37.93 ± 2.92
LGG-407	85.70 ± 0.13	72.85 ± 0.31	15.0	83.17 ± 0.30	62.53 ± 0.43	24.8	10.82 ± 0.36	8.17 ± 0.06	24.5	1.93 ± 0.02	2.84 ± 0.01	47.5	41.10 ± 0.61	39.00 ± 0.78
MGG-351	82.35 ± 0.22	76.90 ± 0.28	6.6	88.68 ± 0.29	77.94 ± 0.54	12.1	5.71 ± 0.01	5.49 ± 0.14	4.0	1.95 ± 0.03	2.49 ± 0.01	28.0	42.33 ± 0.67	35.23 ± 2.66
MCV-1	75.43 ± 0.36	73.95 ± 0.43	2.0	86.93 ± 0.47	81.76 ± 0.79	6.0	7.30 ± 0.05	4.22 ± 0.01	42.2	2.18 ± 0.03	2.59 ± 0.01	18.9	47.80 ± 1.05	51.70 ± 1.12
PAU-911	81.38 ± 0.15	76.80 ± 0.33	5.6	78.40 ± 0.17	75.14 ± 0.09	4.2	3.60 ± 0.09	2.45 ± 0.03	32.0	2.27 ± 0.05	4.05 ± 0.04	78.1	45.63 ± 1.95	36.80 ± 2.34
PDM-11	76.65 ± 0.37	73.59 ± 0.09	4.0	83.13 ± 0.11	67.48 ± 0.41	18.8	10.73 ± 0.14	5.65 ± 0.16	47.3	1.75 ± 0.01	2.57 ± 0.01	46.5	43.23 ± 1.49	43.60 ± 1.33
Phule-m-2	79.72 ± 0.31	76.70 ± 0.40	3.8	90.62 ± 0.12	74.14 ± 0.44	18.2	9.40 ± 0.16	6.07 ± 0.03	35.5	2.34 ± 0.02	3.85 ± 0.03	64.2	38.03 ± 1.15	44.00 ± 1.55
Samrat	78.68 ± 0.11	71.11 ± 0.32	9.6	53.48 ± 0.33	48.14 ± 0.42	10.0	6.75 ± 0.09	5.66 ± 0.22	16.2	1.84 ± 0.01	2.97 ± 0.01	60.8	46.97 ± 1.34	42.00 ±
Sattya	80.87 ± 0.38	78.22 ± 0.29	3.3	48.86 ± 0.30	47.85 ± 0.34	2.1	5.68 ± 0.12	4.25 ± 0.04	25.3	2.56 ± 0.01	3.26 ± 0.02	27.1	47.67 ± 1.06	39.73 ± 2.33
TM-96-2	84.06 ± 0.44	77.50 ± 0.47	7.8	63.97 ± 0.24	53.50 ± 0.27	16.4	5.43 ± 0.01	5.30 ± 0.05	2.3	2.11 ± 0.03	3.18 ± 0.02	50.6	46.50 ± 2.34	41.57 ± 0.72
WGG-37	83.97 ± 0.18	81.96 ± 0.39	2.4	67.49 ± 0.22	54.72 ± 0.17	18.9	10.19 ± 0.09	7.39 ± 0.10	27.5	2.12 ± 0.01	3.72 ± 0.02	75.9	46.40 ± 2.41	42.13 ± 2.30
LGG-460	88.76 ± 0.45	78.31 ± 0.29	11.8	85.23 ± 0.06	68.02 ± 0.32	20.2	9.70 ± 0.02	6.59 ± 0.20	32.1	1.84 ± 0.01	2.41 ± 0.01	31.3	40.37 ± 0.52	55.70 ± 1.84
PDM-54	80.25 ± 0.45	76.25 ± 0.24	5.0	78.33 ± 0.23	59.40 ± 0.47	24.2	6.40 ± 0.25	4.67 ± 0.02	27.0	2.74 ± 0.02	3.15 ± 0.02	15.1	42.83 ± 0.95	41.43 ± 0.72
SML-32	80.27 ± 0.48	74.41 ± 0.34	7.3	84.12 ± 0.19	70.82 ± 0.26	15.8	5.31 ± 0.10	4.75 ± 0.03	10.6	2.16 ± 0.02	2.91 ± 0.01	35.1	44.23 ± 1.50	43.70 ± 1.48
IC-10492	76.59 ± 0.29	73.80 ± 0.18	3.6	81.84 ± 0.14	59.88 ± 0.42	26.8	6.72 ± 0.01	6.09 ± 0.02	9.4	2.18 ± 0.04	2.55 ± 0.03	17.1	39.87 ± 1.82	33.40 ± 1.89
PLM-32	81.15 ± 0.35	71.08 ± 0.44	12.4	85.73 ± 0.24	78.68 ± 0.36	8.2	6.78 ± 0.01	6.18 ± 0.03	8.9	2.09 ± 0.04	4.56 ± 0.02	118.3	45.83 ± 1.70	39.50 ± 1.64
IPM-02-03	79.66 ± 0.23	76.68 ± 0.34	3.7	81.26 ± 0.47	71.53 ± 0.29	12.0	3.90 ± 0.03	3.01 ± 0.02	23.2	2.26 ± 0.01	2.59 ± 0.01	14.6	47.20 ± 2.54	37.30 ± 1.62
LGG-450	80.24 ± 0.18	76.34 ± 0.27	4.8	87.28 ± 0.31	68.74 ± 0.55	21.2	11.87 ± 0.25	3.31 ± 0.16	72.1	2.53 ± 0.02	3.41 ± 0.02	35.0	44.97 ± 1.27	31.57 ± 2.24
PM-5	83.49 ± 0.37	81.16	2.8	84.88 ± 0.34	78.15 ± 0.52	7.9	5.51 ± 0.03	3.17 ± 0.05	42.4	2.67 ± 0.02	3.62 ± 0.01	35.3	43.30 ± 1.54	43.37 ± 1.24
PDM-139	89.10 ± 0.51	74.29 ± 0.34	16.6	76.99 ± 0.09	61.53 ± 0.41	20.1	3.36 ± 0.06	1.61 ± 0.08	52.0	2.18 ± 0.01	2.74 ± 0.03	25.4	43.43 ± 1.79	34.37 ± 0.87
Generalmean	80.76 ± 0.30	75.45 ± 0.31	6.5	79.93 ± 0.277	67.74 ± 0.40	15.1	7.33 ± 0.11	5.05 ± 0.087	28.9	2.19 ± 0.02	2.98 ± 0.02	36.9	43.55 ± 1.68	41.64 ± 1.67
Minimum	75.43	69.96	1.5	48.86	47.85	2.1	3.36	1.61	2.3	1.64	2.07	9.6	36.90	31.57
Maximum	89.10	81.96	16.6	90.62	85.10	31.1	11.87	8.17	72.1	2.74	4.56	118.3	47.80	55.70
Std. dev.	0.52	0.53		0.46	0.68		0.18	0.15		0.03	0.03		2.91	2.91

Con. = Control, Str. = Stress, inc = Increase, dec = Decrease, RWC = Relative water content, MSI = Membrane stability index, CC = chlorophyll content, Std. dev. = Standard deviation.

MSI is the first line of defense in plants under drought stress. Hence, the ability of a plant to maintain membrane stability and integrity would explain its tolerance toward drought (Ahmadizadeh et al., 2011) . Among all varieties, the highest percentage decrease in MSI was recorded for Phule-m-2 (17.75%) and TARM-1 (31.1%), which are considered drought-susceptible. Varieties PLM-32 (0.31%) and Sattya (2.1%) were judged to be drought-tolerant varieties as they showed the lowest percentage decreases in MSI during the vegetative and the reproductive stages, respectively. Relative water content ranged from 61.89% to 84.89% with a mean of 73.82% during the vegetative stage, whereas during the reproductive stage RWC ranged from 69.96% to 81.96% with a mean of 75.45% under drought conditions. It was observed that drought conditions have negative impact on water balance, and hence decrease the water potential of leaves. Therefore, RWC indicates the degree of drought stress, as previous studies have reported that higher decreases in water potential were observed in drought-susceptible varieties than in drought-tolerant varieties (Parvin et al., 2015; Chowdhury et al., 2017) .

In this study, the highest percentage decrease in RWC was observed in varieties TARM-1 (19.52%) and PDM139 (16.6%); the lowest percentage decrease in RWC was recorded in varieties MCV-1 (1.82%) and V. sublobata (1.5%) during the vegetative and reproductive stages, respectively. Among the treatments, irrigated plants maintained the highest RWC and MSI. The lowest MSI (67.7 mS/cm) and lowest RWC (73.8%) were observed in the reproductive and vegetative stages, respectively.

Significant deviation was observed among the varieties since each variety has different ability for absorption and transpiration loss of water through stomata (Baroowa and Gogoi, 2016) . Thus, MSI and RWC are considered to be the key indicators of water status under drought stress (Parvin et al., 2015; Shanazari et al., 2018) .

Biochemical traits such as proline content and protein content exhibited a significant variation (P < 0.001 and P < 0.05) in all 25 mungbean varieties. The mean result showed that under drought conditions, proline content significantly increases and protein content significantly decreases compared to control conditions during both of the development stages. The mean percentage reduction in protein content was higher at the vegetative stage (32.8%, Table 2) than at the reproductive stage (28.9%, Table 3).

At the vegetative stage, the highest protein content was observed in LGG-450 (5.9 µg/mL) and PLM-32 (4.0 µg/ mL) varieties under control and drought conditions, respectively. Similarly, LGG-450 (11.9 µg/mL) and LGG407 (8.2 µg/mL) contained the highest protein content in control and drought conditions during the reproductive stage, respectively. Significant variation in protein reduction occurred in all varieties during stress, probably due to reduced nitrate assimilation at both stages (Yagoob and Yagoob, 2014; Ahmad et al., 2015) . Proline accumulation is the most important physiological index for the plant’s response to drought stress. Change in concentration of proline observed in mungbean varieties exposed to drought stress was higher at the reproductive stage (range: 2.7–4.6 µg/gm; Table 3) than at the vegetative stage (range: 2.7–3.4 µg/gm; Table 2). This is probably because proline accumulation depends upon the leafage, leaf position, and plant age (Bharadwaj et al., 2018) . As with advancement in crop age, leaf water potential decreases under drought stress, and free accumulation of proline occurs. It is believed that proline acts as an osmolyte and protects the plant against low water potential by maintaining osmotic regulation in plant organs (Kabbadj et al., 2017; Silvestre et al., 2017) . In addition to this, proline also plays a major role as an electron receptor and may promote damage repair ability in the plant by increasing antioxidant activity during drought stress (Yaish, 2015) . Proline accumulation was highest in TM-96-2 and PLM-32 at the vegetative stage and the reproductive stage, respectively. Under water stress, proline accumulation was greater than that of other amino acids; therefore, proline can be used as a criterion for screening drought-tolerant varieties (Fahramand et al., 2014) .

**Figure 1:**
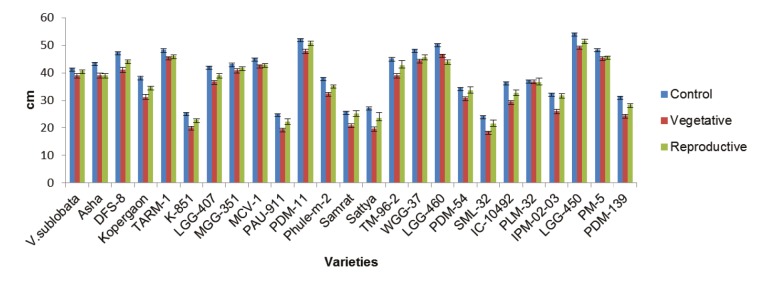
Effect of drought stress on plant height of mungbean varieties during vegetative and reproductive stages; values are mean ± standard error.

**Figure 2:**
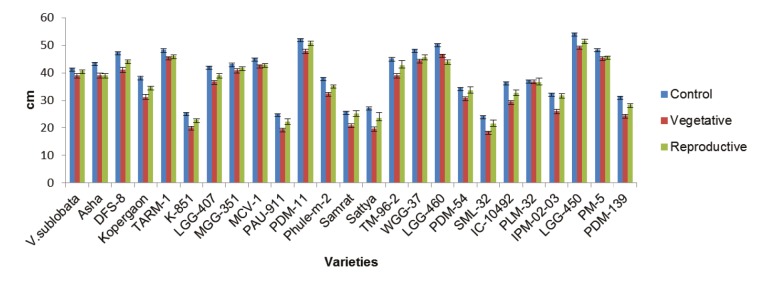
Effect of drought stress on leaf area of mungbean varieties during vegetative and reproductive stages; values are mean ± standard error.

Drought stress had an adverse effect on morphological traits and was significantly affected by exposure to drought stress in both stages (P < 0.001; Table 1). It was observed that irrigated varieties were taller than stressed varieties (Figure 1). Plant height ranged from 24.23 to 54.23 cm with a mean value of 39.52 cm during the control stage. Under drought conditions during the vegetative stage, plant height ranged from 18.60 to 49.43 cm with a mean value of 34.78 cm; under the same conditions during the reproductive stage, plant height ranged from 21.67 to 51.50 cm with a mean value of 39.96 cm. During all stages, LGG-450 maintained the highest plant height. Reduction in leaf area was also observed under drought conditions in both the vegetative (average mean 34.2 cm2) and the reproductive (average mean 45.3 cm2) stages in comparison to control conditions (average mean 66.4 cm2) (Figure 2). The highest leaf area was displayed by PLM-32 and lowest by SML-32 during the vegetative and reproductive stages, respectively. Leaf area plays an important role in drought tolerance in plants.

Morphological acclimatization to drought in mungbean appears to involve reduction of the leaf area. This seems to be a drought-avoiding mechanism, because reducing the leaf area will cause less water loss through transpiration. The decrease in leaf area might be due to the suppression of leaf expansion through reduced cell division owing to loss of cell turgor. Under drought stress, leaf area reductions have been observed in many plant species (Karademir et al., 2012; Avramova et al., 2015; Larkunthod et al., 2018) .

**Figure 3:**
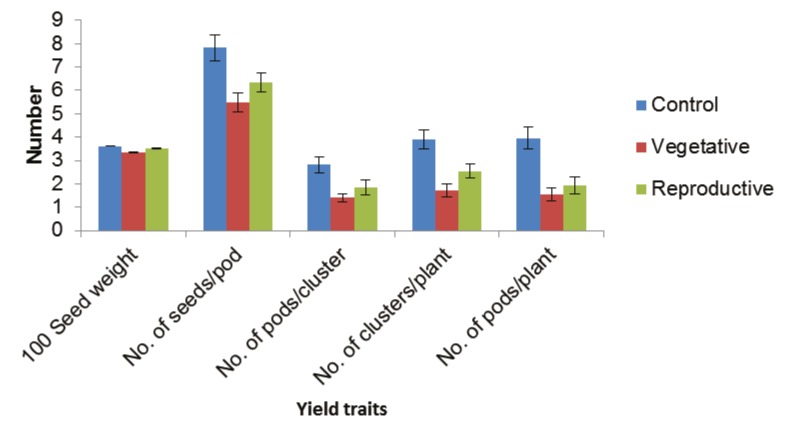
Effect of drought stress on yield components of mungbean varieties during vegetative and reproductive stages; values are mean ± standard error.

SPAD chlorophyll meter readings ranged from 32.3 to 48.8 during the vegetative stage and 31.6 to 55.7 during the reproductive stage. SPAD readings can be used for quick assessment of chlorophyll status (Arunyanark et al., 2008) . Previous studies also support the finding that SPAD values significantly increase under drought stress (Silva et al., 2007; Songsri et al., 2009) . Moreover, reductions in traits like chlorophyll content, plant height, and leaf area may cause low photosynthetic activity, which in turn results in reduced final plant yield. All varieties showed 50% flowering from 35 to 52 days after sowing in irrigated conditions, whereas they flowered within a range of 30 to 50 days under drought conditions. Among all of the varieties, IC-10492 flowered first (30 days), whereas MCV-1 flowered last (50 days). Plant yield was adversely affected, as seen by the reduced number of seeds per pod, seed weight, number of pods per plant, number of clusters per plant, and number of pods per cluster at both the vegetative and reproductive stages (Figure 3).

Overall, drought caused impairments in the processes of cell division and cell expansion and ultimate loss of cell turgor, which are responsible for reduced growth rate, plant height, leaf area, and yield traits. It was observed that irrespective of the varieties, water stress caused a greater adverse effect during the vegetative stage than during the reproductive stage; this correlates with the findings of previous studies (Allahmoradi et al., 2011; Ratnasekera and Subhashi, 2015) . This is probably due to the water absorption capacity being low during the vegetative stage due to a shortage of soil water; consequently, grain yield and growth will be decided by the ability to grow vigorously and accumulate as much dry weight as possible before flowering (Uddin et al., 2013; Baroowa and Gogoi, 2016) .

It was observed that drought stresses markedly affected the physiological characteristics of mungbean varieties; however, the yield reduction was less than what was expected from the impact on physiological characteristics. This variation in morphophysiological traits might be due to the varying nature and duration of stress, as these traits are governed by a large number of genes (Eskandari and Kazemi, 2010; Thirunavukkarasu et al., 2017) . A number of previous studies reported similar morphophysiological variations under variable environmental stress conditions (Naresh et al., 2013; Baroowa and Gogoi, 2016; Raina et al., 2016; Alderfasi et al., 2017) . Under drought stress, PLM-32, MCV-1, TM-96-2 (during the vegetative stage) and the varieties Sattya, V. sublobata, LGG-407, and PLM-32 (during the reproductive stage) maintained high physiological and biochemical traits. Varieties such as MCV-1, PLM-32, and LGG-450 maintained high yield traits under exposure to drought stress and hence are drought-tolerant varieties. Based on the above results, it is suggested that these varieties could be used in breeding programs for better physiological drought tolerance traits.

### 3.2. Cluster analysis and correlation among morphophysiological parameters under drought stress

**Figure 4:**
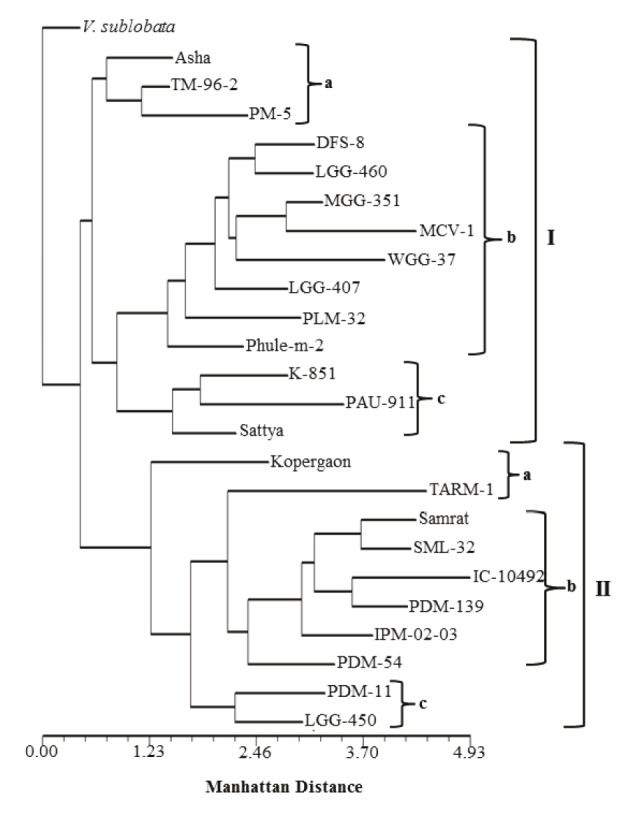
Dendrogram derived from UPGMA cluster analysis using Manhattan distance showing genetic relationship among mungbean varieties.

Based on the morphophysiological data from the control stage, mungbean varieties were grouped into 2 major clusters, with V. sublobata being used for rooting the dendrogram (Figure 4). The Manhattan distance ranged from 0 to 4.93 for all varieties. The first cluster comprised 14 varieties and was further divided into 3 subclusters: Ia, Ib, Ic. Subcluster Ia comprised Asha, TM-96-2, and PM5; subcluster Ib included DFS-8, LGG-460, MGG-351, MCV-1, WGG-37, LGG-407, PLM-32, and Phule-m-2; subcluster Ic contained K-851, PAU-911, and Sattya.

Similarly, the second cluster contained 10 varieties and was also divided into 3 subclusters: IIa, IIb, and IIc. Subcluster IIa consisted of Kopergaon and TARM-1; subcluster IIb included Samrat, SML-32, IC-10492, PDM-139, IPM-0203, and PDM-54; subcluster IIc contained PDM-11 and LGG-450. PAU-911 (1.968) and TARM-1 (2.214) varieties were more distant than other varieties in cluster I and cluster II, respectively.

Correlation analysis revealed that morphophysiological parameters were more strongly correlated with each other during the vegetative stage (Table 4) than during the reproductive stage (Table 5). Overall, proline was negatively associated with protein content (–0.54, –0.43), MSI (–0.50, –0.31), and RWC (–0.42, –0.31), as well as with yield parameters in both the vegetative and the reproductive stages, respectively. This showed that physiological traits are important in sustaining drought tolerance in mungbean. Plant height was not significantly associated with other parameters except for the number of seeds per pod, number of pods per plant, and leaf area. Chlorophyll SPAD value does not show a significant association with other traits in either stage, except with proline and protein content. Leaf area exhibited a positive correlation with RWC (0.57, 0.38), MSI (0.39, 0.31), protein content (0.35, 0.41), and yield components, and was negatively associated with proline content (–0.37, –0.35) in both the vegetative and the reproductive stages. Except for proline content, none of the physiological parameters showed significant association with other physiological parameters during the reproductive stage. Indeed, morphological parameters like yield components, including the number of seeds per pod, number of pods per cluster, number of clusters per plant, and leaf area were more strongly correlated with each other than with the physiological parameters.

**Table 4:** Correlation analysis among morphophysiological and biochemical traits recorded during the vegetative stage under drought stress conditions.

Traits	RWC	MSI	Protein	Proline	CC	LA	PH	SW	NoS/P	NoPo/Cl	NoPo/P	NoCl/P
RWC	1											
MSI	0.34*	1										
Protein	0.35*	0.43**	1									
Proline	–0.42**	–0.50**	–0.54**	1								
CC	–0.13	–0.19	–0.40**	0.37*	1							
LA	0.57**	0.39*	0.35*	–0.37*	–0.16	1						
PH	–0.04	0.17	0.12	–0.06	–0.14	0.42**	1					
SW	0.36*	0.45**	0.14	–0.20	0.09	0.51**	0.22	1				
NoS/P	0.38*	0.49**	0.29	–0.35*	–0.20	0.58**	0.38*	0.39	1			
NoPo/Cl	0.49**	0.50**	0.55**	–0.52**	–0.26	0.65**	0.25	0.39	0.50**	1		
NoPo/P	0.58**	0.50**	0.34*	–0.45**	–0.18	0.76**	0.31*	0.59**	0.60**	0.68**	1	
NoCl/P	0.54**	0.50**	0.34*	–0.39*	–0.15	0.70**	0.24	0.57**	0.65**	0.65**	0.8**	1

* Significant at 5%; ** significant at 1%.

RWC = Relative water content, MSI = Membrane stability index, CC = Chlorophyll content, LA = Leaf area, PH = Plant height, SW = Seed weight, NoS/P = Number of seeds per plant, NoPo/Cl = Number of pods per cluster, NoPo/P = Number of pods per plant, NoCl/P = Number of clusters per plant.

**Table 5:** Correlation analysis among morphophysiological and biochemical traits recorded during the reproductive stage under drought stress conditions.

Traits	RWC	MSI	Protein	Proline	CC	LA	PH	SW	NoS/P	NoPo/Cl	NoPo/P	NoCl/P
RWC	1											
MSI	0.25	1										
Protein	0.23	0.30*	1									
Proline	–0.31*	–0.31*	–0.43**	1								
CC	0.10	0.04	0.07	0.20	1							
LA	0.38*	0.31*	0.41**	–0.35*	0.26	1						
PH	0.24	0.21	0.48**	–0.15	0.08	0.35*	1					
SW	0.18	0.00	0.30*	–0.20	0.29	0.42**	0.18	1				
NoS/P	0.34*	0.17	0.33*	–0.44**	0.15	0.46**	0.33*	0.40**	1			
NoPo/Cl	0.28	0.50**	0.45**	–0.37*	0.12	0.44**	0.11	0.11	0.30*	1		
NoPo/P	0.48**	0.41**	0.40*	–0.40**	0.23	0.68**	0.23	0.39*	0.43**	0.54**	1	
NoCl/P	0.47**	0.41**	0.28	–0.47**	0.30*	0.62**	0.19	0.39*	0.53**	0.50**	0.73**	1

* Significant at 5%; ** significant at 1%.

RWC = Relative water content, MSI = Membrane stability index, CC = Chlorophyll content, LA = Leaf area, PH = Plant height, SW = Seed weight, NoS/P = Number of seeds per plant, NoPo/Cl = Number of pods per cluster, NoPo/P = Number of pods per plant, NoCl/P = Number of clusters per plant.

Overall, in terms of studied morphophysiological and biochemical traits, large variations were observed in mungbean varieties at both stages under drought stress.

However, the vegetative stage proved to be more susceptible to drought stress. Therefore, removal of irrigation during the vegetative stage can be cost effective. It was also demonstrated that RWC, MSI, proline, and protein content could be used as quick screening criteria for drought tolerance. Furthermore, the importance of biophysiological traits in sustaining drought tolerance is supported by the correlation analysis. Based on these criteria, the varieties V. sublobata, MCV-1, PLM-32, LGG-407, LGG-450, TM-962, and Sattya varieties were considered drought-tolerant at both stages among all varieties. Therefore, knowledge of these morphophysiological and biological responses and their correlation in mungbean under drought conditions might contribute to ongoing studies of drought resistance in mungbean.

## Acknowledgments

The authors thank the Indian Council of Agricultural Research and the Director, National Bureau of Plant Genetic Resources, New Delhi for the facilities provided for the study. The first author acknowledges the fellowship from The Department of Biotechnology, Government of India for the PhD program.
